# A new species of *Lycodon* Boie, 1826 (Serpentes, Colubridae) from northern Vietnam

**DOI:** 10.3897/zookeys.875.35933

**Published:** 2019-09-10

**Authors:** Helen Y. Janssen, Cuong T. Pham, Hanh Thi Ngo, Minh Duc Le, Truong Q. Nguyen, Thomas Ziegler

**Affiliations:** 1 AG Zoologischer Garten Köln, Riehler Strasse 173, D-50735 Cologne, Germany; 2 Institute of Zoology, University of Cologne, Zülpicher Strasse 47b, D-50674 Cologne, Germany; 3 Institute of Ecology and Biological Resources, Vietnam Academy of Science and Technology, 18 Hoang Quoc Viet, Cau Giay, Hanoi, Vietnam; 4 Faculty of Biology, Hanoi University of Science, Vietnam National University, 334 Nguyen Trai Road, Hanoi, Vietnam; 5 Faculty of Environmental Sciences, Hanoi University of Science, Vietnam National University, 334 Nguyen Trai Road, Hanoi, Vietnam; 6 Central Institute for Natural Resources and Environmental Studies, Hanoi National University, 19 Le Thanh Tong, Hanoi, Vietnam; 7 Department of Herpetology, American Museum of Natural History, Central Park West at 79th Street, New York, New York 10024, USA; 8 Graduate University of Science and Technology, Vietnam Academy of Science and Technology, 18 Hoang Quoc Viet, Cau Giay, Hanoi, Vietnam

**Keywords:** Cao Bang Province, *Lycodon pictus* sp. nov., morphology, phylogeny, taxonomy

## Abstract

A new species of the genus *Lycodon* is described from Cao Bang Province, Vietnam, based on three individuals with distinct differences in morphology and molecular data. The new species is differentiated from its congeners by a combination of the following characters: dorsal scales in 17-17-15 rows, smooth throughout; supralabials usually eight (rarely nine); infralabials ten; one elongated loreal on each side, in contact with the eye; precloacal plate single; ventral scales 212–218 (plus one or two preventral scales); subcaudals 90 or 91; maxillary teeth 13 or 14; dorsal surface of body with 28 or 29 light body bands; dorsal surface of tail with 13 cream bands, forming a distinct blotch in the vertebral region. Based on phylogenetic analyses of mitochondrial cytochrome *b* sequence data, the new species is recovered as the sister species to a clade containing *L. multizonatus* and *L. liuchengchaoi* with strong support from the Bayesian analysis. The new species is at least 7.5% divergent from other species within this clade in uncorrected pairwise distance calculated using a fragment of more than 1000 bp of the mitochondrial cytochrome *b.* This discovery increases the number of *Lycodon* species known from Vietnam to 16.

## Introduction

The genus *Lycodon* Boie, 1827 is one of the most diverse genera of colubrid snakes, with 61 currently recognised species (Uetz et al. 2019, Luu et al. 2019). Recent phylogenetic studies showed that the genera *Dinodon*, *Dryocalamus* and *Lepturophis* nested within *Lycodon* and suggested to place them into the genus *Lycodon* sensu lato (Guo et al. 2013; Siler et al. 2013; Figueroa et al. 2016). The members of this genus have a broad distribution from eastern Iran to southern China and Japan, southward to the Philippines as well as the Indo-Australian Archipelago (Lanza 1999; Siler et al. 2013; Neang et al. 2014). Six species of *Lycodon* have been described in the last five years, namely *L. zoosvictoriae* Neang, Hartmann, Hun, Souter & Furey, 2014 from Cambodia; *L. cavernicolus* Grismer, Quah, Anuar, Muin, Wood & Nor, 2014 from Malaysia; *L. sidiki* Wostl, Hamidy, Kurniawan & Smith, 2017 from Indonesia; *L. banksi* Luu, Bonkowski, Nguyen, Le, Calame & Ziegler, 2018 from Laos, *L. namdongensis* Luu, Ziegler, Ha, Le & Hoang, 2019 from Vietnam and *L. gibsonae* Vogel & David, 2019 from Thailand. From Vietnam, fifteen species of *Lycodon* have been reported to date, comprising *L. capucinus* (Boie, 1827), *L. cardamomensis* (Daltry & Wüster, 2002), *L. davisonii* (Blanford, 1878), *L. fasciatus* (Anderson, 1879), *L. flavozonatus* (Pope, 1928), *L. futsingensis* (Pope, 1928), *L. laoensis* Günther, 1864, *L. meridionalis* (Bourret, 1935), *L. namdongensis* Luu, Ziegler, Ha, Le & Hoang. 2019, *L. paucifasciatus* Rendahl in Smith, 1943, *L. rosozonatus* (Hu & Zhao, 1972), *L. rufozonatus* Cantor, 1842, *L. ruhstrati abditus* Vogel, David, Pauwels, Sumontha, Norval, Hendrix, Vu & Ziegler, 2009, *L. septentrionalis* (Günther, 1875), and *L. subcinctus* Boie, 1827 (Uetz et al. 2019, Luu et al. 2019).

Our recent field surveys in the Ha Lang and Trung Khanh districts, Cao Bang Province, northern Vietnam, revealed a snake population that was referable to the genus *Lycodon* based on the following characters: nostril enlarged; robustly arched upper maxillary bone with an inward curve in the anterior part; anterior and posterior maxillary teeth interrupted by a diastema; dorsal scales smooth or weakly keeled, in 17 rows anteriorly and at midbody, and posteriorly 15 rows (Lanza 1999; Grismer et al. 2014). However, the series of three individuals from Cao Bang were morphologically distinct from other named species. These morphological results were further corroborated by the analysis of a fragment of the mitochondrial cytochrome *b* gene, and so herein we describe the population from Cao Bang Province, northern Vietnam, as a new *Lycodon* species.

## Materials and methods

### Sampling

The field surveys were led by TQN in October 2011 and from April to May 2012. The collected specimens were euthanised with ethyl-acetate, fixed in approximately 85% ethanol for 10 hours, and subsequently transferred to 70% ethanol for permanent storage. Liver tissue samples were preserved separately in 95% ethanol. The specimens were deposited in the collections of the Institute of Ecology and Biological Resources (**IEBR**), Hanoi, Vietnam and of the Zoologisches Forschungsmuseum Alexander Koenig (**ZFMK**), Bonn, Germany.

### Morphological analysis

Identification of sex was performed by dissection (inspection of gonads and presence of hemipenes). Maxillary teeth were counted by dissecting the right maxilla for teeth / sockets. Scalation and maxillary teeth number were examined with a binocular dissecting microscope. Measurements were taken following Ziegler et al. (2018) with a measuring tape to the nearest 1 mm.

Abbreviations of morphological characters are as follows:

**SVL** Snout-vent length (from tip of snout to vent);

**TaL** tail length;

**TaL / TL** ratio of tail length / total length;

**TL** total length;

**DSR** dorsal scale rows number at one head length posterior to the head – number of dorsal scale rows at midbody – number of dorsal scale rows at one head length anterior to the vent;

**SL** supralabials (counted on upper lips);

**SL / orbit** number of supralabials entering orbit;

**IL** infralabials (counted on lower lips);

**Lor** loreals;

**Lor / eye** loreal scale touching the eye (yes or no);

**PreOc** preoculars;

**PostOc** postoculars;

**Atem** number of anterior temporals;

**PTem** number of posterior temporals;

**BodySc** scalation of the body (keeled or smooth);

**PreVen** number of preventral scales;

**Ven** number of ventral scales;

**SubC** number of subcaudal scales;

**Prec** precloacal (or cloacal) plate (single or divided);

**Teeth max** number of maxillary teeth / alveoli.

Scale counts were taken following Vogel et al. (2009). Ventral scales (Ven) were counted according to Dowling (1951). Bilateral scale counts were given as left / right.

Comparisons were mainly based on the data provided by Boulenger (1893), Pope (1928), Smith (1943), Leviton (1965), Ota and Ross (1994), Manthey and Grossmann (1997), Lanza (1999), Vogel et al. (2009), Vogel and David (2010) and Neang et al. (2014), with additional references provided in the comparisons and legends of the tables. Additionally, studied specimens are listed in the Appendix 1.

### Molecular data and phylogenetic analyses

Representative taxa of the genus *Lycodon* were included in the study. Sequences of the species were downloaded from GenBank. Two samples of the population from Cao Bang Province (ZFMK 93746, ZFMK 93747) were incorporated in the analysis. *Boiga cynodon* (Boie 1827) and *Dipsadoboa flavida broadleyi* (Broadley & Stevens, 1971) were used as outgroups based on Figueroa et al. (2016).

DNA was extracted using DNeasy Blood and Tissue kit (Qiagen, Germany) following the manufacturer’s instructions. A fragment of the mitochondrial cytochrome *b* gene was amplified using the primer pair L14910 (5’–GACCTGTGATMTGAAAACCAYCGTTGT-3’) and H16064 (5’– CTTTGGTTTACAAGAACAATGCTTTA-3’; Burbrink et al. 2000). Extracted DNA was amplified using HotStarTaq Mastermix (Qiagen, Germany) with 21 µl volume consisting of 10 µl of Mastermix, 5 µl of water, 2 µl of each primer at 10 pmol/ml and 2 µl of DNA. PCR conditions were 95 °C for 15 minutes to activate the Taq, with 40 cycles of 95 °C for 30 s, 45 °C for 45 s, 72 °C for 60 s, and a final extension at 72 °C for six minutes. The mitochondrial cytochrome *b* gene was utilised in this study because it has been widely used in previous molecular analyses of *Lycodon* (e.g., Guo et al. 2013, Siler et al. 2013), and has been shown to be informative in revealing new species of *Lycodon* (e.g., Grismer et al. 2014, Luu et al. 2018, 2019).

PCR products were visualised using gel electrophoresis through a 2% low melting-point agarose gel stained with ethidium bromide. Successful amplifications were purified to eliminate PCR components using GeneJET^TM^ PCR Purification kit (ThermoFisher Scientific, Lithuania). Purified PCR products were sent to FirstBase (Malaysia) for sequencing.

The obtained sequences were aligned in ClustalX 1.8.3 (Thompson et al. 1997) using the default settings. Data were analysed using maximum parsimony (MP) as implemented in PAUP*4.0b10 (Swofford 2001) and Bayesian inference (BI) as implemented in MrBayes v3.2 (Ronquist et al. 2012). Settings for these analyses followed Le et al. (2006), except that the number of generations in the Bayesian analysis was increased to 1×10^7^. For the maximum likelihood (ML) analysis, we used IQ-TREE v.1.6.7.1 (Nguyen et al. 2015) with a single model and 10,000 ultrafast bootstrap replications. For ML and BI, the optimal model for nucleotide evolution was set to TrN+I+G by Modeltest v3.7 (Posada and Crandall 1998). For BI, the analysis was conducted with a random starting tree and run for 10^7^ generations. Four Markov chains, one cold and three heated (utilising default heating values), were sampled every 1000 generations. Log-likelihood scores of sample points were plotted against generation time to detect stationarity of the Markov chains. The burn-in value was set to 26 in the BI analysis, as -ln*L* scores reached stationarity after 26,000 generations in both runs. Two independent analyses were run simultaneously. Nodal support was evaluated using Bootstrap replication (BP) as estimated in PAUP*4.0b10 and IQ-TREE v1.6.7.1 and posterior probability (PP) in MrBayes v3.2. BP ≥ 70 and PP ≥ 95% are regarded as strong support for a clade. Uncorrected pairwise distances (*p*) were calculated in PAUP*4.0b10.

## Results

### Molecular data and phylogenetic analyses

**Figure 1.**
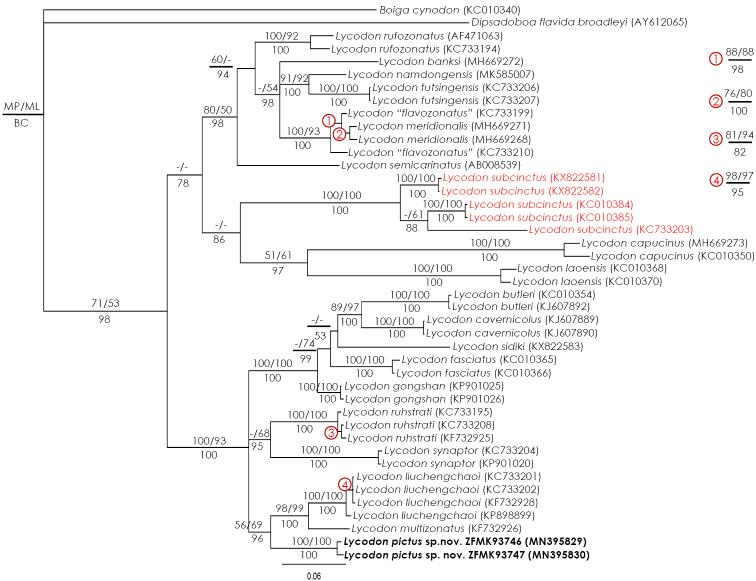
Bayesian cladogram based on the partial mitochondrial cytochrome *b* gene of snakes belonging to the genus *Lycodon*. Numbers above and below branches are bootstrap values of MP/ML analyses (≥ 50%) and Bayesian posterior probabilities (BC), respectively.

The final matrix consisted of 1011 bp aligned characters and the alignment contained no gaps. In total, 404 characters were found to be parsimony informative. MP analysis resulted in five most parsimonious trees having 1662 steps (CI = 0.41, RI = 0.72). Our tree topologies are very similar to those recovered by Guo et al. (2015) and Luu et al. (2018). The new species was recovered to be the sister species to a clade containing *L. multizonatus* + *L. liuchengchaoi*, with strong support in BI (PP = 96), but weak support in MP and ML (BP_MP_ = 56, BP_ML_ = 69) (Fig. 1). The new species has an uncorrected *p*-distance of at least 7.5% and 8.1% from *Lycodon liuchengchaoi* Zhang, Jang, Vogel & Rao, 2011 and *L. multizonatus* Zhao & Jiang, 1981, respectively.

#### Lycodon pictus sp. nov.

Taxon classificationAnimaliaSquamataColubridae

F0B264E9EDEE5AAEB2E773234A138CAD

http://zoobank.org/FEA7DFD1-BF41-4608-A477-93861FF13AD4

**Figure 2.**
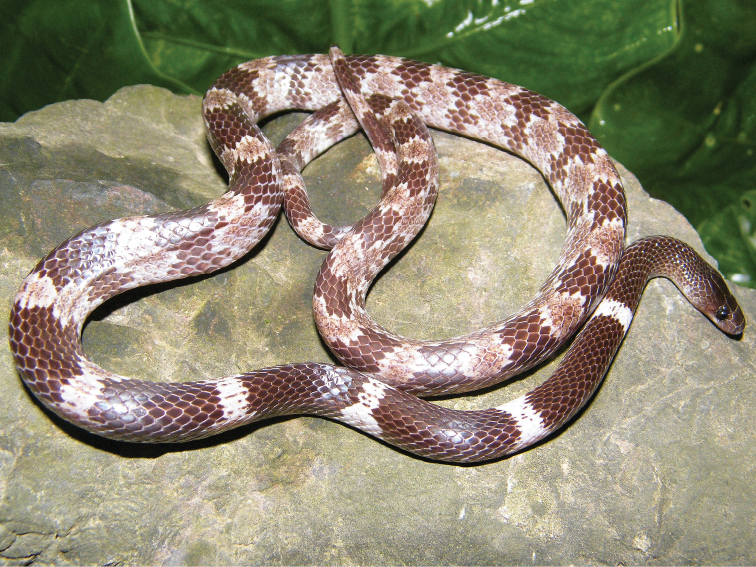
Holotype of *Lycodon pictus* sp. nov. (IEBR 4166) in life. Photograph T. Lehmann.

**Figure 3.**
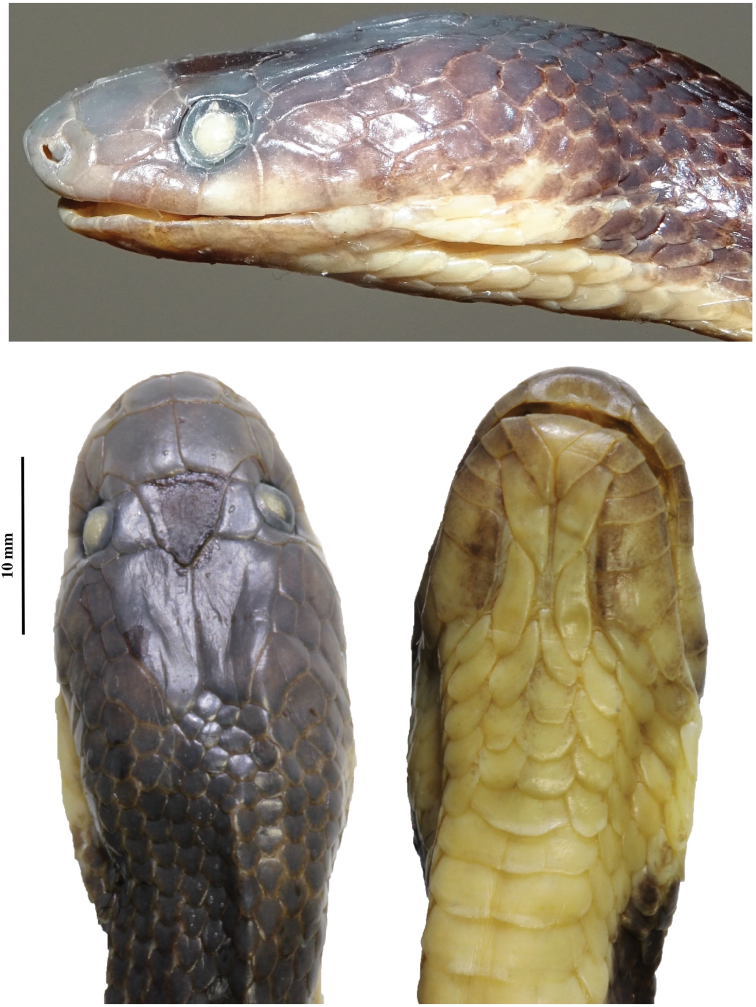
Head views of *Lycodon pictus* sp. nov. (IEBR 4166) in preservative (scale bar refers to head in dorsal view). Photographs T. Ziegler (upper), C.T. Pham (lower).

**Figure 4.**
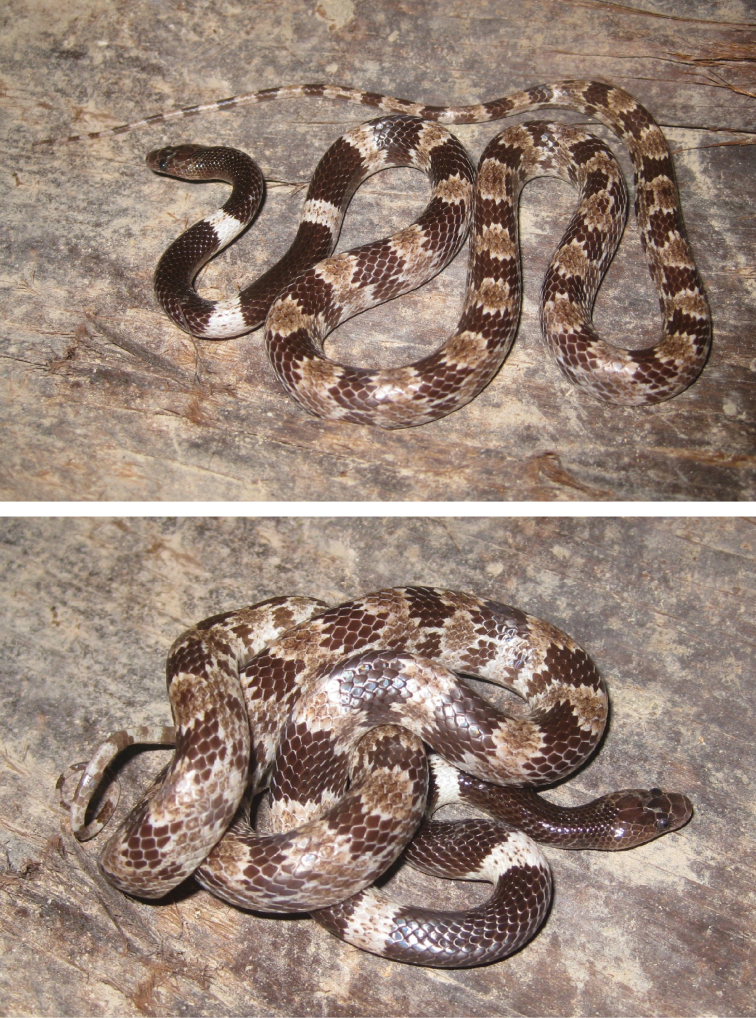
Female paratype of *Lycodon pictus* sp. nov. (ZFMK 93746) in life. Photographs T.Q. Nguyen.

**Figure 5.**
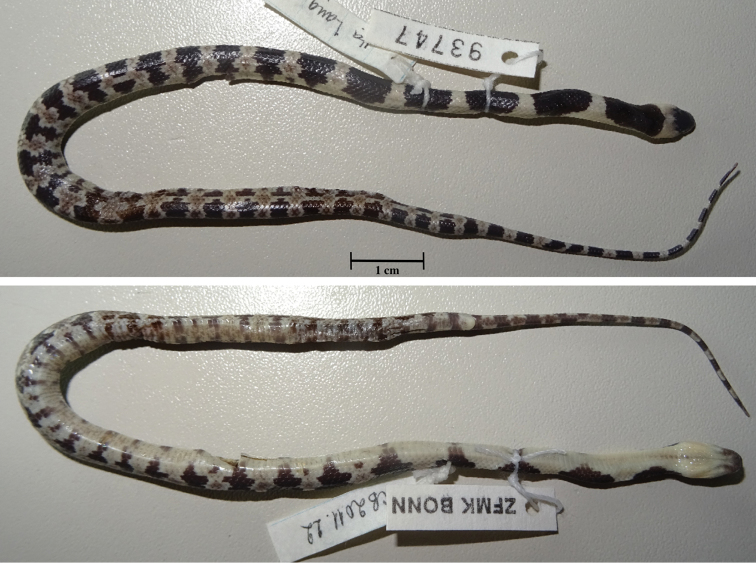
Juvenile paratype of *Lycodon pictus* sp. nov. (ZFMK 93747) in preservative (upper, dorsal view; lower, ventral view). Photographs T. Ziegler.

**Figure 6.**
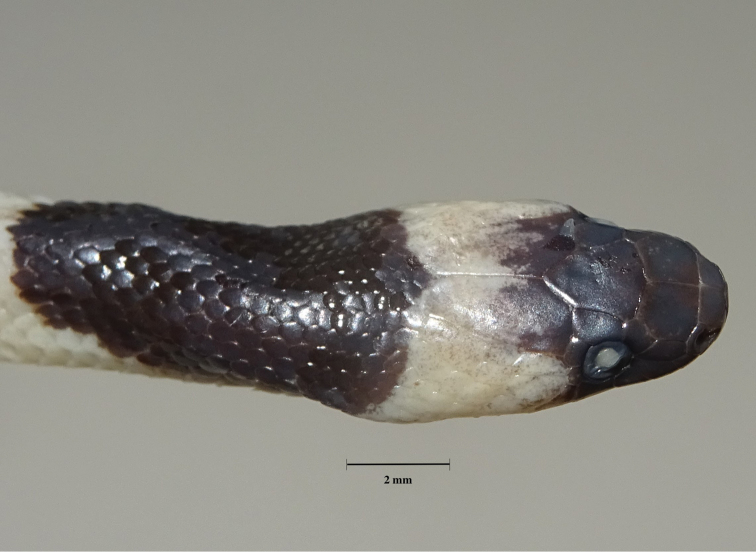
Dorsal head and neck pattern of the juvenile paratype of *Lycodon pictus* sp. nov. (ZFMK 93747) in preservative. Photograph T. Ziegler.

Figs 2 , 3 , 4 , 5 , 6

##### Holotype.

IEBR 4166 (field number CB 2012.97), adult male, collected on 18 April 2012 by TQN et al. (altitude 701 m a.s.l.), Trung Khanh District, Cao Bang Province.

##### Paratypes.

ZFMK 93747, juvenile, collected on 15 October 2011 by TQN et al. (altitude 588 m a.s.l.), Ha Lang District, Cao Bang Province; ZFMK 93746, adult female, collected on 10 April 2012 by TQN et al., Ha Lang District, Cao Bang Province.

##### Diagnosis.

*Lycodon pictus* sp. nov. can be differentiated from its congeners by the following morphological characters: dorsal scales in 17–17–15 rows, all smooth; supralabials usually eight (rarely nine); infralabials ten; one elongated loreal on each side, in contact with the eye; precloacal plate single; ventral scales 212–218 (plus one or two preventral scales); subcaudals 90 or 91; a total length of 597+ mm in males and 543 mm in females; tail / total length ratio 0.211–0.215; maxillary teeth 13 or 14; dorsal surface of body with 28 or 29 light body bands; dorsal surface of tail with 13 cream bands forming a distinct blotch in the vertebral region; ventral surface of body and tail mostly cream with the dark body bands in part extending towards the venter, sometimes forming complete dark bands around the body.

##### Description of the holotype.

Head elongate, moderately distinct from the neck, rather flattened, longer than wide, narrow anteriorly; nostril lateral, located in the middle of the nasal; eye large, pupils vertically elliptic; rostral triangular, much broader than high, hardly visible from above; nasal divided into two scales by a vertical ridge along posterior edge of nostril; two internasals, anteriorly rounded, slightly wider than high, bordered by two large, pentagonal prefrontals posteriorly; frontal single, enlarged, pentagonal to hexagonal, narrowed posteriorly; parietals longer than wide, in contact with each other medially, with upper anterior and posterior temporals, paraparietal laterally and four nuchal scales posteriorly; paraparietals elongated, anterior part widened; loreal 1/1, elongate, not entering orbit; supralabials 8/8, first and second in contact with nasal, third to fifth entering orbit, sixth largest; infralabials 10/10, first pair in broad contact with each other, first to fifth in contact with anterior pair of chin shields; anterior and posterior pairs of chin shields elongate, of the same size and shape, second pair not meeting in midline; preocular 1/1; postoculars 2/2, lowermost smaller, bordering anterior temporals; anterior temporals 2/2, posterior temporals 3/3, upper ones thinner than lower ones. Left maxilla arched, with an angular apex, distinctly bent inwards anteriorly. A total of 13 maxillary teeth or teeth alveola, with the following formula: five small anterior teeth, with the last two ones being somewhat enlarged + two strongly enlarged teeth, thick, and not much curved + a wide gap, somewhat wider than the length of the largest teeth + four small teeth + a small gap + two enlarged posterior teeth.

Body elongate, SVL 488 mm; TaL > 109 mm (tail tip lost); preventral 1, ventrals 212, from behind neck region distinctly notched laterally; subcaudals > 54 (tail tip lost), paired; precloacal plate single; DSR 17-17-15, all smooth; the vertebral scales not enlarged; DSR reduction from 17 to 15 at the position of ventral 150.

##### Coloration in preservative.

Head, neck, and dorsal surface of body brownish black; light body bands beginning after 1.5 times the head length behind the head, in total 29 transverse light bands on body and at least nine light bands on tail; the first four body bands yellowish cream, and distinctly widened towards the venter, increased in size posteriorly; a dark mottling in the vertebrate region more prominent posteriorly; the subsequent light body bands with two distinct indentations on each side, fused in the middle in the last third of the body. In dorsal view the light bands forming a distinct blotch in the vertebral region, with a dark centre and a lighter frame; laterally, the middle part of the light bands forming blotches, but wider and with an extended dark centre, fused laterally in the last third part of the body; the lower and widest part of the light body bands with a dark small blotch in the centre in the anterior part of body; the light bands on the tail with a blotch like pattern in the vertebral region, but less pronounced than that on body, and one light blotch at the lateral side of tail, widened towards the venter, with a dark centre; ventral surface of head and neck yellowish cream, belly cream and greyish cream in the last third part of body and on lower tail surface; the dark dorsal bands (28 on body and at least nine on tail) in part extending towards the venter (most prominent in the anterior five dark body bands), not forming complete dark bands around the body, but complete on the tail; lateral side of the head dark above and light below, with the lighter pattern beginning in the supralabial region; tip of lower jaw and infralabial region in part greyish; dorsal surface of the head and upper head sides a bit paler than the remaining head dorsum.

##### Hemipenis.

Hemipenes elongated, not fully everted, not turgid. Truncus without spines. Spine ornamentation starting at truncus region with somewhat enlarged, medium sized spines. Apex with microspines. Sulcus stretches in the middle to apex. Apex not fully everted, ending somewhat widened with an oblique opening, with microspines inside, pointing to the not fully everted condition of the outer genital organ.

##### Variations.

In the juvenile ZFMK 93747, the number of supralabials on the left side is nine, with fourth to sixth entering the orbit. The loreal does not touch the eye on the right side. The lower anterior temporal scale is not touching the postocular scale on the left side. In general, the coloration is more intense in the juvenile. The creamy pattern on the posterior third of the body sides is connected by a horizontal cream-colored stripe. It has a yellowish cream band on the head that reaches from SL 5 behind the jaws and distinctly lightens the posterior half of the head but does not touch the frontal. In the juvenile, the banded pattern is more simple, consisting of dark bands which narrow towards the venter and light bands which widen towards the venter and bear a dark pattern and a more or less distinct dark blotch at the lower side (see Fig. 5).

In the female ZFMK 93746, the lower anterior temporal scale is not touching the postocular scale on the right side. For measurements and scalation data of the examined specimens see Table 1.

**Table 1.** Sex, measurements (in mm), scalation data, and coloration pattern of *Lycodon pictus* sp. nov. For abbreviations see Materials and methods. Key: asterisk (^*^) lower Atem not touching PostOc; plus sign (+) tail tip lost.

	Holotype IEBR 4166	Paratype ZFMK 93746	Paratype ZFMK 93747
Sex	male	female	juvenile
TL	597	543	237
SVL	488	426	187
TaL	109+	117	50
TaL/TL	–	0.215	0.211
Teeth max	13	13	14
SL	8/8	8/8	9/8
SL/orbit	3–5	3–5	4–6/3–5
IL	10/10	10/10	10/10
PreOc	1/1	1/1	1/1
PostOc	2/2	2/2	2/2
Lor	1/1	1/1	1/1
Atem	2/2	2^*^/2	2/2^*^
PTem	3/3	3/3	3/3
DSR	17-17-15	17-17-15	17-17-15
PreVen	1	2	1
Ven	212	216	218
Prec	single	single	single
Subc	54+	91	90
BodySc	smooth	smooth	smooth
Dark bands on body	28	29	28
Light bands on body	29	29	28
Dark bands on tail	9	13	13
Light bands on tail	9+	13	13

##### Dentition.

Female ZFMK 93746 and juvenile ZFMK 93747: Left maxilla arched, with an angular apex, distinctly bent inwards anteriorly. A total of 13 (in female) or 14 (in juvenile) maxillary teeth or teeth alveola, with the following formula: five small anterior teeth, with the last two ones being somewhat enlarged + two strongly enlarged teeth, thick, and not much curved + a wide gap, somewhat wider than the length of the largest teeth + four small teeth + a small gap + two enlarged posterior teeth in the female and three posterior teeth in the juvenile, with the anterior two ones enlarged.

##### Comparisons.

In our phylogenetic analysis, *Lycodon pictus* sp. nov. is most closely related to *L. liuchengchaoi* and *L. multizonatus*. From *L. liuchengchaoi*, the new species differs in terms of body scalation (all smooth in the new species vs. feebly keeled in several median rows in *L. liuchengchaoi*), head scalation (ten infralabials vs. 7–9) and dentition (13 or 14 maxillary teeth vs. 8 or 9). In addition, the new species differs from the latter in having 28 or 29 cream body bands (vs. 40 yellow rings on the body in *L. liuchengchaoi*) (Zhang et al. 2015).

The new species differs from *L. multizonatus* by having more maxillary teeth (13 or 14 vs. 10 or 11 in *L. multizonatus*), more infralabials (10 vs. 8) and a single precloacal plate (vs. divided). In addition, the new species differs from the latter in terms of body scalation (minimum 212 ventrals and minimum 90 subcaudals vs. 190–195 ventrals and 68–75 subcaudals in *L. multizonatus*). Furthermore, *L. pictus* sp. nov. has fewer light body bands (28 or 29 vs. 55–73 in *L. multizonatus*) (Lei et al. 2014).

From its Vietnamese congeners, the new species can be differentiated as follows: *Lycodon pictus* sp. nov. differs from *L. capucinus* in having a single precloacal plate (vs. divided), a loreal touching the eye (vs. not in contact with the eye), in having more ventrals (minimum 212 vs. 182–211) and more subcaudals (90 or 91 vs. 59–74), and in terms of dorsal pattern (banded vs. reticulated) (Luu et al. 2019).

*Lycodon pictus* sp. nov. differs from *L. cardamomensis* in terms of dorsal scalation (17-17-15 smooth DSR vs. 19-17-15 weakly keeled DSR), in having a loreal in contact with the eye (vs. separated) and in having 28 or 29 light body bands (vs. 12-14 pinkish orange body bands) (Daltry and Wüster 2002, Do et al. 2017).

*Lycodon pictus* sp. nov. differs from *L. davisonii* in having 17 midbody dorsal scale rows (vs. 13 midbody dorsal scale rows), fewer ventral scales (maximum 218 vs. 235–265), more infralabials (10 vs. 8) and the absence of preocular (vs. present). In addition, the new species differs from the latter in having a different dorsal pattern (28 or 29 cream bands on body vs. 36 white rings on the body) (Blanford 1878, Boulenger 1893).

*Lycodon pictus* sp. nov. differs from *L. fasciatus* in having smooth dorsal scales (vs. keeled) and more maxillary teeth (13 or 14 vs. 11). Additionally, the colour pattern of *Lycodon pictus* sp. nov. differs in being dark brownish black with light body bands turning into a marbling posteriorly, whereas *L. fasciatus* is black or purplish black above with yellowish cross-bars of irregular outline and has a dark median stippling (Pope 1928, Smith 1943). Werner (1922) described *Dinodon yunnanensis* from Yunnan Fu, now Kunming, Yunnan Province, southwestern China. This species was synonymized with *Lycodon fasciatus* by Pope (1935: 188), but according to Vogel and David (2010), this taxon might be a distinct species (see also Vogel and David 2019). *Lycodon pictus* sp. nov. differs from *Dinodon yunnanensis* Werner, 1922 in having more ventrals (minimum 212 vs. 193), more subcaudals (90 or 91 vs. 66), more infralabials (10 vs. 9) and more light body bands (28 or 29 vs. 23) (Werner 1922, Vogel and David 2010, Vogel and David 2019).

*Lycodon pictus* sp. nov. differs from *L. flavozonatus* in terms of dorsal scalation (smooth vs. keeled), in having more subcaudals (90 or 91 vs. 80–88), the loreal in contact with the eye in *Lycodon pictus* sp. nov. (vs. separated in *L. flavozonatus*) and in coloration pattern (brownish black with 28 or 29 cream body bands and 9–13 light bands on the tail vs. black with 68 yellow body bands and 21 on the tail) (Pope 1928, Vogt in Pope 1928).

*Lycodon pictus* sp. nov. differs from *L. futsingensis* in having more ventrals (minimum 212 vs. 193–208) and more subcaudals (minimum 90 vs. 72–87). Additionally, the loreal does not enter the orbit in *L. futsingensis*, whereas it enters the orbit in *Lycodon pictus* sp. nov. (Vogel et al. 2009).

*Lycodon pictus* sp. nov. differs from *L. laoensis* in having a single precloacal plate (vs. divided), more ventral scales (minimum 212 vs. 163–192), more subcaudal scales (minimum 90 vs. 60–76), an elongated loreal scale in contact with the orbit (vs. separated) and cream body bands (vs. yellow) (Grismer et al. 2014, Neang et al. 2014).

*Lycodon pictus* sp. nov. differs from *L. meridionalis* in having smooth dorsals (vs. feebly keeled in 10–12 median rows), a lower ventral scale count (maximum 218 vs. 227–240) and fewer subcaudals (maximum 91 vs. 96–106). In addition, the new species differs in having cream body bands (vs. yellow thin crossbars) (Gawor et al. 2016 and examined ZFMK specimens, see Appendix 1).

*Lycodon pictus* sp. nov. differs from *L. namdongensis* in having more subcaudals (90 or 91 vs. 85) and the loreal in contact with the eye (vs. separated from the eye in *L. namdongensis*). The new species also differs in coloration pattern (brownish black with 28 or 29 light bands on the body vs. grey with 23 cream cross rings on the body in *L. namdongensis*), and in having irregular bands turning into a marbling posteriorly (vs. clearly demarcated cross bands on the body) (Luu et al. 2019).

*Lycodon pictus* sp. nov. differs from *L. paucifasciatus* in terms of dorsal scalation (17-17-15 smooth DSR vs. 19-(19-17)-15 DSR, the upper one or two plus vertebral row distinctly keeled) and fewer ventral scales (maximum 218 vs. 219–222). In addition, the new species has a loreal entering the eye (vs. separated) and 28 or 29 light body bands (vs. 14–25 beige or dirty cream body bands) (Vogel et al. 2009).

*Lycodon pictus* sp. nov. differs from *L. rosozonatus* in having 17-17-15 smooth DSR (vs. 19-19- 15(17) keeled DSR), fewer ventral scales (maximum 218 vs. 221–234) and a loreal in contact with the eye (vs. separated). In addition, the new species has cream body bands (vs. pinkish red) (Hu et al. 1975, Neang et al. 2014).

*Lycodon pictus* sp. nov. differs from *L. rufozonatus* in having a loreal in contact with the eye (vs. separated), smooth dorsal scales (vs. feebly keeled in the posterior body part), and in coloration pattern (28 or 29 cream body bands vs. 44–52 light red body bands) (Zhao 2006, Luu et al 2018).

*Lycodon pictus* sp. nov. differs from *L. ruhstrati abditus* in having smooth dorsals (vs. 7–8 dorsal scale (including vertebral) rows keeled), an elongated loreal in contact with the eye (vs. separated), and in having irregular bands turning into a marbling posteriorly (vs. clearly demarcated cross bands on the body) (Vogel et al. 2009).

*Lycodon pictus* sp. nov. differs from *L. septentrionalis* by its smooth dorsal scales (vs. 7–9 median rows feebly keeled), 10 infralabials (vs. 7 or 8), and the loreal entering the orbit (vs. separated in *L. septentrionalis*). In addition, the new species differs in having cream irregular bands on a brown body (vs. white narrow bands on a black body forming complete annuli) (Günther 1875, Boulenger 1893, Neang et al. 2014).

*Lycodon pictus* sp. nov. differs from *L. subcinctus* in having 10 infralabials (vs. 8 or 9), one preocular (vs. preocular absent), smooth dorsal scales (vs. feebly keeled) and 28 or 29 cream bands on the body and 9–13 on the tail (vs. 9–15 bands on the body and none on the tail) (Boulenger 1893, Neang et al. 2014).

*Lycodon pictus* sp. nov. differs from *L. ophiophagus*, a species from southern Thailand but with similar scalation, in having a loreal entering the eye (vs. separated) and in dorsal colour pattern (28 or 29 light bands on a brown body vs. 20 or 21 white bands on a dark body) as well as and in having irregular bands turning into a marbling posteriorly (vs. clearly demarcated cross bands on the body) (Vogel et al 2009).

For additional measurements, dentition, and scalation data see Tables 2–8.

##### Distribution.

**Figure 7.**
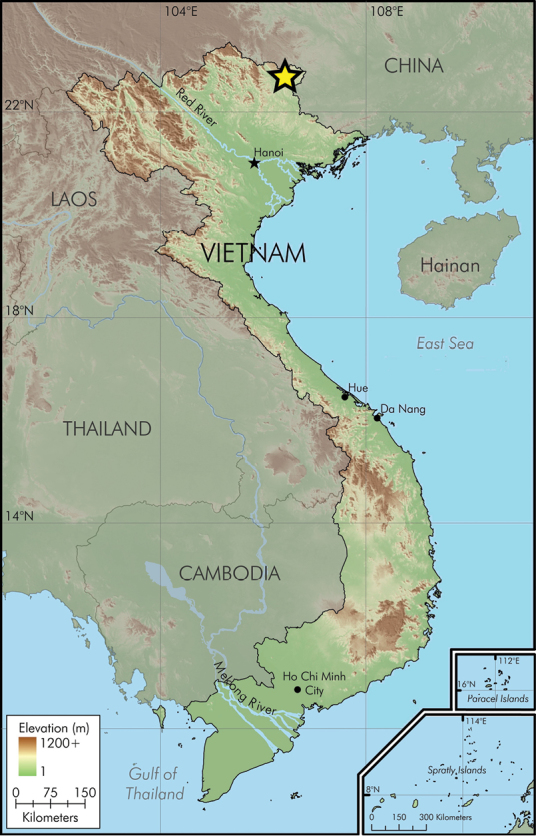
Map showing the type locality of *Lycodon pictus* sp. nov. in Cao Bang Province.

*Lycodon pictus* sp. nov. is currently known only from Ha Lang and Trung Khanh districts, Cao Bang Province, northern Vietnam (Fig. 7).

##### Etymology.

The name of the species *pictus* means painted or decorated in Latin and refers to its unique dorsal colour pattern.

##### Natural history.

**Figure 8.**
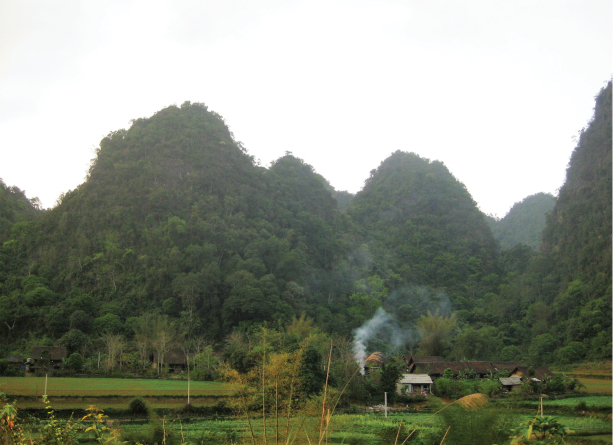
Habitat of *Lycodon pictus* sp. nov.: the female paratype (ZFMK 93746) was found in Ha Lang District, Cao Bang Province.

*Lycodon pictus* sp. nov. seems to be closely associated with karst environment. Specimens were found at night between 19:00 and 23:00, on forest paths or on the ground near cave entrances. The surrounding habitat was secondary karst forest, consisting of medium and small hardwood trees mixed with shrubs and vines. Air temperature was 23.4–29.6°C and humidity was 66–79%. Other reptiles were also found at the site, including *Acanthosaura lepidogaster* (Cuvier, 1829), *Gekko adleri* Nguyen, Wang, Yang, Lehmann, Le, Ziegler & Bonkowski, 2013, *Goniurosaurus luii* Grismer, Viets & Boyle, 1999, *Lycodon futsingensis* (Pope, 1928), and *Protobothrops trungkhanhensis* Orlov, Ryabov & Nguyen, 2009 (Fig. 8).

**Table 2.** Measurements (in mm), dentition, and scalation data of *Lycodon* species from Vietnam. Data taken from Blanford (1878), Pope (1928), Smith (1943), Boulenger (1893), Daltry and Wüster (2002), Jackson and Fritts (2004), Vogel et al. (2009), Neang et al. (2014), Do et al. (2017), and Luu et al. (2019); distinguishing characters are marked in bold. For abbreviations see Materials and methods.

	*Lycodon pictus* sp. nov.	*L. capucinus*	*L. cardamomensis*	*L. davisonii*	*L. fasciatus*	*L. flavozonatus*	*L. futsingensis*	*L. laoensis*
TL	597	816	896	920 ♂	894	1440 ♂,1210 ♀	850 ♂ 773 ♀	470
Teeth max	13 or 14	**15**	**10–12**		**11**	13	12–15	
SL	8	**9 or 10**	8	**7**	8	8	8 (7)	**9 or 10**
SL/orbit	3–5	3–5	3–5	3–4	3–5	3–5	3–5 (4–5; 2–4;4–6)	3–5
IL	10	9 or 10	10	**8**	9 (8, 10)	10	10 (9, 11)	10
PreOc	1	1	1	**0**	1	1	1	1
PostOc	2	2	2 or 3	1 or 2	2	2	2 (3)	2 (3)
Lor	1	1	1	1	1	1	1	1
Lor/eye	yes	**no**	**no**	yes	yes	**no**	**no**	**no**
Atem	2	2	2	1 or 2	2	2	2 (1)	2
PTem	3	3	2 or 3	**2**	**2**	2 or 3	3 (2)	3
DSR	17-17-15	17-17-15	**19**-17-15	**13**	17-17-15	17-17-15	17-17 (16)- 15	17-17-15
Ven	212-218	**182-211**	215-228	**233-265**	182-225	211-221 ♂ 212-218 ♀	**193-204 ♂, 198-208**♀	**163-192**
Prec	single	**divided**	single	single	single	divided/ single	single	**divided**
Subc	90 or 91	**59-74**	87-93	90-108	65-94	**81-88** ♂, **80-84** ♀	**72-87 ♂, 78-85** ♀	**60-76**
BodySc	smooth	**weakly keeled**	**weakly keeled**	smooth	**keeled**	**7 rows feebly keeled at midbody**	smooth	smooth

**Table 3.** Measurements (in mm), dentition, and scalation data of *Lycodon* species from Vietnam (continuation of Table 2). Data from Günther (1875), Boulenger (1893), Hu et al. (1975), Zhao (2006), Vogel et al. (2009), Guo et al. (2013), Neang et al. (2014), Gawor et al. (2016), Luu et al. (2018), Luu et al. (2019), and based on examined specimens from ZFMK (see Appendix 1). Key: distinguishing characters are marked in bold; * Luu et al. (2019) mention 240 ventrals as maximum for *L. rufozonatus*; however, that is a transcription error from Bourret (1935) who gave 204 as the maximum number of ventrals. For abbreviations see Materials and methods.

	*L. meridionalis*	*L. namdongensis*	*L. paucifasciatus*	*L. rosozonatus*	*L. rufozonatus*	*L. ruhstrati abditus*	*L. septentrionalis*	*L. subcinctus*
TL	1139	723	763	1060	1234	964	1163	1000
Teeth max	**11**	**12**	**11 or 12**	12 or 13	11–13	11–13	**7**	8–14
SL	8	8	8	8	8	8	8	8
SL/orbit	3–5	3–5	3–5		3–5	3–5	3–5	3–5 (3–6)
IL	10	10	10		10 (9)	10 (9,11)	**7 or 8**	**8 or 9**
PreOc	1	2/1	1	1	1	1	1	**0**
PostOc	2	3	2	2	2	2	2	2 or 3
Lor	1	1	1	1	1	1	1	1
Lor/eye	**no**	**no**	**no**	**no**	**no**	**no**	**no**	yes
Atem	2	2	2	2	2	2 (1)	2	**1**
PTem	3	**2**	3	3	3	3 (2)	3	**2**
DSR	17-17-15	17-17-15	**19**- (19-17)- 15	**19**-**19**- 15(17)	(19-17)-17-15	17-17-15	17-17-15	17-17-15
Ven	**227-240**	218	**219-222**	**221-234**	184-225*	197-229	202-224	192-230
Prec	divided	single	single		single	single	single	divided (rarely single)
Subc	**96-106**	**85**	90-92		53-98	90-103	83-104	60-91
BodySc	**dorsals feebly keeled in 10-12 median rows**, smooth in outer rows	smooth	**upper 1 or 2 dorsal scale row(s) plus vertebral row distinctly keeled**	**weakly keeled**	**feebly keeled in the posterior body part**	smooth (DSR 1-6), **distinctly keeled (DSR 7-8 and vertebral row)**	**7 or 9 median rows feebly keeled**	**feebly keeled**

**Table 4.** Measurements (in mm), dentition and scalation data of *Lycodon* species from China, Laos and Cambodia. Data taken from Zhao and Jiang (1981), Boulenger (1893), Vogel and David (2010)Vogel and Luo (2011), Zhang et al. (2011), Vogel et al. (2012), Lei et al. (2014), Neang et al. (2014), Zhang et al. (2015), Ganesh and Vogel (2018), and Luu et al. (2018). Key: plus sign (+) tail incomplete; distinguishing characters are marked in bold. For abbreviations see Materials and methods.

	*L. aulicus*	*L. banksi*	*L. davidi*	*L. gongshan*	*L. liuchengchaoi*	*L. multizonatus*	*L. synaptor*	*L. zoosvictoriae*
TL	719	465 +	389.5	963 ♂	676	505	487	520.7
Teeth max			**11**		**8 or 9**	**10 or 11**	**10**	**9**
SL	**9 (8 or 10)**	8	8	8	7 or 8	8 (rarely 7)	8	8
SL/orbit	3–5	3–5	3–5	3–5	3–5	3–5	3–5	3–5/4–5
IL	10 or 11	10	10	**8**	**8 (7,9)**	**8 (7)**	**8**	10
PreOc	1	1	1	1	1	0 or 1	1	1 or 2
PostOc	2	2	2	2	2	2	2	2
Lor	1	1	1	1	1	1	1	1
Lor/eye	**no**	yes	**no**	yes	yes	yes	**no**	**no**
Atem	2	2	2	2	1-3	2 (1)	2	2
PTem	3	3	2 or 3	2 or 3	1-3	3 (2)	**2**	**2**
DSR	17-17-15	17-17-15	17-17-15	17-17-15	17-17-15	17-17-15	15 or 17-17-15	17-17-15
Ven	180-215	**241**	**224** ♂	210–216 ♂, 215 ♀	190-228	**190-195**	**201-203** ♀	213 ♀
Prec	**divided**	single	single	single	**divided**	**divided**	single	single
Subc	**57-78**	26 +	**99** ♂	**95 or 96 ♂, 92** ♀	**68-77**	**68-75**	**68 or 69** ♀	**85** ♀
BodySc	smooth and glossy	smooth **(six central DSR of posterior 1/3 feebly keeled)**	**middorsal scale rows slightly keeled**, outermost rows entirely smooth	**upper dorsal rows 6–12 and vertebral row keeled**	**feebly keeled in median rows**	smooth	**6-7 upper rows and vertebral row feebly keeled**	**weakly keeled**

**Table 5.** Measurements (in mm), dentition, and scalation data of *Lycodon* species from Thailand and Myanmar. Data taken from Günther (1864), Boulenger (1893), Boulenger (1900), Smith (1943), Lanza (1999), Slowinski et al. (2001), Daltry and Wüster (2002), Vogel et al. (2009), Bahuguna and Bhuta (2010), Grismer et al. (2014), Luu et al. (2018), and Vogel and David (2019); distinguishing characters are marked in bold. For abbreviations see Materials and methods.

	*L. albofuscus*	*L. butleri*	*L. effraenis*	*L. gibsonae*	*L. gracilis*	*L. jara*	*L. kundui*	*L. ophiophagus*	*L. subannulatus*	*L. zawi*
TL	1480	876	700	906	533	535 ♂, 550 ♀		909	684	480
Teeth max	**12**			13	**9**			11-13	**8 or 10**	**12**
SL	8	8 or 9	**9**	8	8	8 or 9	**7**	8	**7**	8 or 9
SL/orbit	3–5	3–5	3–5	3–4 and 3–5	3–4	3–5	3–4	3–5	3–4	3–5
IL		9 or 10	10 or 11	10				10	**8**	9 or 10
PreOc	1	1	1	1	2	1		1	1	1
PostOc	2	2	2-3	2	2	2	2	2	2	2 (1)
Lor	1	1	**0**	1	1 (united with lower PreOc)	1	1	1	1	1
Lor/eye	**no**	yes	**no Lor**	yes	yes	**no**	**no**	**no**	yes	**no**
Atem	2	2	2	2	2	1 or 2	**1**	2	2	2 (3)
PTem	**2**	**2**	2 or 3	3	3	2 or 3	**2**	3	**2**	3 (4)
DSR	17	19 (17 in Boulenger 1900)	17	17-17-15	**15**	17-17-15	**15**-**15**-15	17-17-15	**15**-**15**-15	17-17-15
Ven	**241**	**220-227**	215-228	**223-226** ♂	234	**167-188**	**186**	211-212	**225-244**	**179-194** ♂, **207** ♀
Prec	**divided**	single	single	single	single	**divided**	divided (entire in Lanza 1999)	single	single	**divided**
Subc	**155-208**	81-96	72-99	91-92 ♂	**81-83**	**52-74**	**70**	87-90	**93-111**	**45-75** ♂
BodySc	**keeled**	**keeled**	smooth	**upper 3 or 4 rows keeled**	**keeled**	smooth	smooth	smooth	**keeled**	smooth

**Table 6.** Measurements (in mm), dentition and scalation data of *Lycodon* species from India. Data taken from Boulenger (1893), Wall (1906), Smith (1943), Taylor (1950), Captain (1999), Vijayakumar and David (2005), Mukherjee and Bhupathy (2007), Mistry et al. (2007), Vogel and Luo (2011), Vogel and Harikrishnan (2013), Ganesh and Vogel (2018), and Melvinselvan et al. (2018); distinguishing characters are marked in bold. For abbreviations see Materials and methods.

	*L. anamallensis*	*L. flavicollis*	*L. flavomaculatus*	*L. gammiei*	*L. hypsirhinoides*	*L. mackinnoni*	*L. nympha*	*L. striatus*	*L. tiwarii*	*L. travancoricus*
TL	522	543	520	1150	717 ♂, 563 ♀	365	574	432	790	600 ♂, 625 ♀
Teeth max							**8–10**			
SL	**9**	**9**	**9**	8 (7,9)	**9**	8 (7)	7 (8 or 6)	**9**		**9**
SL/orbit	3–5	3–5	3–5	3–5 (3–4)	3–5	3–5	3–4	3–5		3–5
IL	10 or 11	11	10		10	**8**		**11**		
PreOc	1	1	1	1	1	1	1 or 2	1		1
PostOc	2 or 3	2	2	2 (1)	2	2	2	2		2
Lor	1/1 (except Holotype 2/2)	1	1	1	1	1 (0 in Wall 1906)	1	1		1
Lor/eye		**no**	**no**	**no**	**no**	**no**	yes	**no**		**no**
Atem	2	2 (3)	2 (1)	2 or irregular	2	1 or 2	2	2 rarely 1		2 or 3
PTem	3+4	3 (rarely 2)	3 (rarely 2)	2 or irregular	3	2 or 3	2 or 3	3 rarely 2		3
DSR	17-17-15	17-17-15	17-17-15	17-17 (19)-15	17-17-15	17-17-15	**13-13-13**	17-17-15	?-17-15	17-17-15
Ven	**174–186 ♂, 186–204** ♀	210-224	**165-183**	205-220	**188–202 ♂, 199–210** ♀	**163- 187**	**200-243**	**153-178**	218-237	**176-206**
Prec	divided (except holotype)	**divided**	**divided**	single	**divided**	**divided**	**divided**	**divided**	**divided**	single
Subc	**63–73** ♂, **60–74** ♀	**65-72**	**53-63**	**98-111**	**68–75 ♂, 61–68** ♀	**48-56**	**65-88**	**42-66**	61-102	64-76
BodySc	smooth	smooth **with single apical pit**	smooth	**9 dorsal rows keeled**, 5 rows at each side smoot	smooth	smooth	**keeled**	smooth		smooth

**Table 7.** Measurements (in mm), dentition and scalation data of *Lycodon* species from the Philippines. Data taken from Griffin (1909), Taylor (1922), Leviton (1965), Ota and Ross (1994), Lanza (1999), Gaulke (2002), and Gaulke et al (2003); distinguishing characters are marked in bold. For abbreviations see Materials and methods.

	*L. alcalai*	*L. bibonius*	*L. chrysoprateros*	*L. dumerilii*	*L. fausti*	*L. ferroni*	*L. muelleri*	*L. philipinus*	*L. solivagus*	*L. tessellatus*
TL	787 ♂	511	727	547 ♂, 521 ♀	337	382	302 ♂, 204 ♀	486	946	900
Teeth max	11–13	11–14	11–13	13–15	13	**12**	14–15	**8**	11–13	
SL	**9**	7–9	**9**	11–13	**9**	**10**	**9**	**7**	**9**	8 or 9
SL/orbit	4–5	3–5 (4–5)	3–5	4–5	4–5	4–6	4–5	3–4	4–5	4–5
IL	10	9 or 10	10	9 or 10	9 or 10	10	10	**7**	10	
PreOc	**2**	**2**	**2**	1 or 2	**2**	**2**	1 or 2	0 or 1	**2**	1
PostOc	**3**	2 or 3	2 or 3	2	**3**	2	2 or 3	2 or 3	2 or 3	2
Lor	1	1	1	1	1	1	1	1	1	1
Lor/eye	**no**	**no**	**no**	**no** (only when fused with PreOc)		**no**	**no**	yes	**no**	**no**
Atem	2	2+3	2+3+4	2	2	2	2	2	2	2
PTem	3	2+3+3	2+3+3	3	3 or 2	3+4	3+4	3	3	2 or 3
DSR	**19**-17-15	**19**-17-15	**19**-17-15	**19**-17-15	**19**- 17- 15	**19**-17-15	19-17-15	**15**	**19**-17-15	17 or 21
Ven	**203-207**	204-212	**186-194**	195-221	207 or 215	**203**	205-213	216-225	**198-203**	**222-232**
Prec	single	single	single	single	single	single	single	single	single	**divided**
Subc	**108-126**	**110-120**	**111-117**	**111-120**	**135-148**	**109**	**112-117**	87-99	**112-115**	**56**
BodySc	smooth	smooth	smooth		smooth	smooth		smooth	smooth	smooth

**Table 8.** Measurements (in mm), dentition and scalation data of *Lycodon* species from Sri Lanka, Malaysia, Japan and Indonesia. Data taken from Boulenger (1893), Stejneger (1907), Smith (1943), Vogel et al. (2009), Grismer et al. (2014), and Wostl et al. (2017); distinguishing characters are marked in bold. For abbreviations see Materials and methods.

	*L. carinatus*	*L. cavernicolus*	*L. multifasciatus*	*L. orientalis*	*L. semicarinatus*	*L. sidiki*	*L. stormi*	*L. tristrigatus*
TL	730	508.2	700	660	1100	715	597	360
Teeth max				**10 or 11**		**7**		**8 or 10**
SL	8 or 9	**9 or 10**		8	8	8	8	**7**
SL/orbit	3-5	4-6		3-5	3-5	3-5	3-4	3-4
IL		10 or 11				10/9		
PreOc	1	1		**0**	1	**0**	1	**0**
PostOc	2	2		2	2	2	2	2
Lor	1	1		1	1	1	1	1
Lor/eye	**no**	yes	**no**	yes	**no**	yes	**no**	yes
Atem	2	3 (2)		2	2	2	**1**	2
PTem	2 or 3	3 or 4		3	3	**2**	3	2 or 3
DSR	17 or 19-**19**-17	17-17-15	17-17-?	17	17	17-17-15	**19**	**15**
Ven	**185-202**	**245 ♂, 232** ♀	**232-237 ♂ 229-235** ♀	**200-208**	211-234	**195**	217	**224**
Prec	single	single		**divided**	single	**divided**	single	single
Subc	**51-64**	**113 ♂, 92** ♀	**115-119 ♂ 106- 117** ♀	**68-74**	**65-105**	**85**	**75**	**86**
BodySc	**strongly keeled**	**the 8 medial rows weakly keeled**	**keeled**	**scales with a very faint keel along their anterior half**	**scales keeled along anterior half** (4 outer rows smooth, other with a feeble though distinct keel on the basal half of each scale)	**keeled**	smooth	**keeled**

## Discussion

Our phylogenetic analyses reveal *Lycodon pictus* sp. nov. to be the sister taxon to a clade containing *L. multizonatus* and *L. liuchengchaoi* from China, but only with strong statistical support in the BI. The new species differed from the latter by at least 7.5% in uncorrected pairwise sequence distance. There has been some taxonomic confusion in the genus *Lycodon*. Two of the *L. liuchengchaoi* sequences (KC733201, KC733202) in the phylogenetic tree had previously been identified as *L. fasciatus*, but the phylogenetic analysis by Guo et al. (2015) correctly assigned them to *L. liuchengchaoi. Lycodon* “*flavozonatus*”, on the other hand, appears to be paraphyletic with *Lycodon* “*meridionalis*” (MH669271, MH669268). Moreover, the *Lycodon subcinctus* species group is likely to contain cryptic diversity. In terms of uncorrected pairwise genetic distance of populations within this species group, two samples (GenBank numbers KX822581 and KX822582) are approximately 9.1–9.2% divergent from KC733203 and 6.3–6.5% from KC010384 and KC010385. The latter two clades differ by approximately 8.0% from each other. These issues need to be further investigated in future studies.

This new discovery increases the number of *Lycodon* known from Vietnam to 16, of which nine are confined to karst formations, underlining the importance of this habitat in promoting reptile speciation (Luu et al. 2018). Although Vietnam is located in the region with one of the most extensive limestone outcrops in the world (Day and Urich 2000) many of the areas are still poorly surveyed, and likely contain a high level of cryptic diversity. Recent studies show that this habitat harbours a significant portion of endemic diversity in the region and should be protected from anthropogenic threats (Clement et al. 2006, Nguyen et al. 2015, Luu et al. 2016).

## Supplementary Material

XML Treatment for Lycodon pictus

## Appendix 1

**Comparative specimens examined**

*Lycodon fasciatus*. Vietnam: Quang Binh Province (ZFMK 86448)

*Lycodon fasciatus*. Vietnam: Quang Binh Province (ZFMK 86449)

*Lycodon fasciatus*. Vietnam: Quang Binh Province (ZFMK 86450)

*Lycodon futsingensis*. Vietnam: Cao Bang (IEBR 4165)

*Lycodon futsingensis*. Vietnam: Cao Bang (IEBR 4170)

*Lycodon futsingensis*. Vietnam: Vinh Phuc (ZFMK 89385)

*Lycodon laoensis*. Cambodia: Phnom Penh (ZFMK 54886)

*Lycodon laoensis*. Vietnam: Dong Nai (ZFMK 88928)

*Lycodon meridionalis*. Vietnam: Quang Ninh (ZFMK 95193)

*Lycodon meridionalis*. Vietnam: Hai Phong (ZFMK 94906)

*Lycodon meridionalis*. Vietnam: Bac Giang (ZFMK 89389)

*Lycodon meridionalis*. Vietnam: Vinh Phuc (ZFMK 89225)

*Lycodon paucifasciatus*. Vietnam: Quang Binh (ZFMK 86452)

*Lycodon paucifasciatus*. Vietnam: Quang Binh (ZFMK 80661)

*Lycodon paucifasciatus*. Vietnam: Quang Binh (ZFMK 80662)

*Lycodon subcinctus*. Indonesia: Bali (ZFMK 95499)

*Lycodon subcinctus*. Vietnam: Dong Nai (ZFMK 91899)
